# Utility of pre-procedural [^99m^Tc]TcMAA SPECT/CT Multicompartment Dosimetry for Treatment Planning of ^90^Y Glass microspheres in patients with Hepatocellular Carcinoma: comparison of anatomic versus [^99m^Tc]TcMAA-based Segmentation

**DOI:** 10.1007/s00259-024-06920-6

**Published:** 2024-09-27

**Authors:** Marnix Lam, Etienne Garin, Paul Haste, Alban Denys, Brian Geller, S. Cheenu Kappadath, Cuneyt Turkmen, Daniel Y. Sze, Hamad Saleh Alsuhaibani, Ken Herrmann, Marco Maccauro, Murat Cantasdemir, Matthew Dreher, Kirk D. Fowers, Vanessa Gates, Riad Salem

**Affiliations:** 1 Department of Radiology and Nuclear Medicine, University Medical Center Utrecht, Huispostnummer E01.132. Postbus 85500, Utrecht, 3508 GA The Netherlands; 2 Nuclear Medicine Department, Eugene Marquis Center, Rennes, France; 3 Department of Clinical Radiology and Imaging Sciences, Indiana University School of Medicine, Indianapolis, IN USA; 4 Department of Radiology and Interventional Radiology, Lausanne University Hospital CHUV, University of Lausanne, Lausanne, Switzerland; 5 Department of Radiology, University of Florida, Gainesville, FL USA; 6 Department of Imaging Physics, University of Texas MD Anderson Cancer Center, Houston, TX USA; 7 Department of Nuclear Medicine, Istanbul Faculty of Medicine, Istanbul University, Istanbul, Turkey; 8 Division of Interventional Radiology, Stanford University, Stanford, CA USA; 9 Department of Radiology, King Faisal Specialist Hospital, Riyadh, Saudi Arabia; 10 Department of Nuclear Medicine, University of Duisburg-Essen, and German Cancer Consortium (TKTK)-University Hospital Essen, Essen, Germany; 11 Nuclear Medicine Unit, Fondazione IRCCS Istituto Nazionale Dei Tumori, Milan, Italy; 12 Division of Interventional Radiology, Istanbul Florence Nightingale Hospital, Istanbul, Turkey; 13 Boston Scientific Corporation, Marlborough, MA USA; 14 Department of Radiology, Northwestern Feinberg School of Medicine, Chicago, IL USA; 15 Department of Radiology and Nuclear Medicine, University Medical Center Utrecht, Heidelberglaan 100, Utrecht, 3584 CX The Netherlands

**Keywords:** Radioembolization, Yttrium-90, Dosimetry, Hepatocellular carcinoma, Segmentation

## Abstract

**Purpose:**

Pre-treatment [^99m^Tc]TcMAA-based radioembolization treatment planning using multicompartment dosimetry involves the definition of the tumor and normal tissue compartments and calculation of the prescribed absorbed doses. The aim was to compare the real-world utility of anatomic and [^99m^Tc]TcMAA-based segmentation of tumor and normal tissue compartments.

**Materials and methods:**

Included patients had HCC treated by glass [^90^Y]yttrium microspheres, ≥ 1 tumor, ≥ 3 cm diameter and [^99m^Tc]TcMAA SPECT/CT imaging before treatment. Segmentation was performed retrospectively using dedicated dosimetry software: (1) anatomic (diagnostic CT/MRI-based), and (2) [^99m^Tc]TcMAA threshold-based (i.e., using an activity-isocontour threshold). CT/MRI was co-registered with [^99m^Tc]TcMAA SPECT/CT. Logistic regression and Cox regression, respectively, were used to evaluate relationships between total perfused tumor absorbed dose (TAD) and objective response rate (ORR) and overall survival (OS). In a subset-analysis pre- and post-treatment dosimetry were compared using Bland-Altman analysis and Pearson’s correlation coefficient.

**Results:**

A total of 209 patients were enrolled. Total perfused tumor and normal tissue volumes were larger when using anatomic versus [^99m^Tc]TcMAA threshold segmentation, resulting in lower absorbed doses. mRECIST ORR was higher with increasing total perfused TAD (odds ratio per 100 Gy TAD increase was 1.22 (95% CI: 1.01–1.49; *p* = 0.044) for anatomic and 1.19 (95% CI: 1.04–1.37; *p* = 0.012) for [^99m^Tc]TcMAA threshold segmentation. Higher total perfused TAD was associated with improved OS (hazard ratio per 100 Gy TAD increase was 0.826 (95% CI: 0.714–0.954; *p* = 0.009) and 0.847 (95% CI: 0.765–0.936; *p* = 0.001) for anatomic and [^99m^Tc]TcMAA threshold segmentation, respectively). For pre- vs. post-treatment dosimetry comparison, the average bias for total perfused TAD was + 11.5 Gy (95% limits of agreement: -227.0 to 250.0) with a strong positive correlation (Pearson’s correlation coefficient = 0.80).

**Conclusion:**

Real-world data support [^99m^Tc]TcMAA imaging to estimate absorbed doses prior to treatment of HCC with glass [^90^Y]yttrium microspheres. Both anatomic and [^99m^Tc]TcMAA threshold methods were suitable for treatment planning.

**Trial registration number:**

NCT03295006.

**Supplementary Information:**

The online version contains supplementary material available at 10.1007/s00259-024-06920-6.

## Introduction

Options for management of hepatocellular carcinoma (HCC) include both systemic and locoregional techniques. Trans-arterial radioembolization using glass [^90^Y]yttrium (^90^Y)-microspheres (*T*heraSphere™; Boston Scientific Corporation, Marlborough, MA, USA) offers a well-established, efficient locoregional treatment option. Multiple studies have reported favorable clinical outcomes and improved survival using radioembolization for HCC [1–3].

Utilization of radioembolization is optimized by increasing tumor absorbed dose (TAD) while minimizing the normal tissue absorbed dose (NTAD) [2–5]. Early dosimetry recommendations have evolved from a typically lobar perfused volume with a single compartment dosimetry target of 120 Gy to personalized dosimetry techniques using either more selective infusions of ≥ 400 Gy average absorbed dose in ≤ 2 Couinaud liver segments (i.e., ablative radiation segmentectomy) [3, 6], or multicompartment dosimetry [7, 8] aiming for an effective TAD and safe NTAD in larger perfused volumes [2, 9]. Multicompartment dosimetry recognizes that tumor and normal liver tissue have distinct levels of absorbed dose with important impact on efficacy and toxicity. The evaluation of both TAD and NTAD allows for a balance in safety (by minimizing NTAD) and efficacy (by increasing TAD), making it particularly beneficial for less selective infusions [2, 10].

Previous publications have highlighted the ability of pre-treatment technetium-99m macroaggregated albumin ([^99m^Tc]TcMAA ) to act as a surrogate for glass ^90^Y-microspheres for predictive dosimetry in HCC patients [11–15]. Pre-treatment [^99m^Tc]TcMAA Single Photon Emission Computed Tomography (SPECT) analysis allows for predictable TADs administered to achieve improved tumor response, and to better define the NTAD effect [2, 12, 16, 17]. Current clinical guidelines state that because of the potential for mismatch between pre-treatment [^99m^Tc]TcMAA prescribed dose simulation and actual post-treatment delivered absorbed doses, absorbed doses should be calculated both pre- and post-treatment. Although planning dosimetry can be performed according to a mean absorbed dose approach (i.e., assuming homogeneous dose distribution within the volume of interest), the more accurate multi-compartment dose evaluation is recommended to follow [18, 19], especially for larger heterogenous tumors. Fully corrected [^99m^Tc]TcMAA SPECT/computed tomography (CT), ^90^Y SPECT/CT and ^90^Y Positron Emission Tomography (PET)/CT are considered optimal acquisition methodologies. Non-attenuation corrected [^99m^Tc]TcMAA SPECT may be used as an alternative only if SPECT/CT is unavailable [2, 7, 12].

Improved clinical outcomes have been reported when TAD is increased by using multicompartment dosimetry-based treatment planning [2, 13, 20]. Previously published data from the TARGET study reported that median overall survival (OS) increased from the lowest to middle to highest TAD subgroups by anatomic (diagnostic CT/MRI-based) segmentation, with the median OS of the highest subgroup (36.7 months for > 300 Gy) being more than double that of the lowest TAD subgroup (16.1 months for < 200 Gy). No association was found between higher NTAD and occurrence of at least grade 3 hyperbilirubinemia. Moreover, overall incidence of hyperbilirubinemia was low at 4.8% [1]. Other potentially NTAD associated toxicity events also failed to demonstrate an association with NTAD, mostly due to low incidence. The lack of association supports the ability to balance efficacy and safety using multicompartment dosimetry and suggests that further optimization is achievable [1].

Advanced dosimetry-based treatment planning is based on the ability of [^99m^Tc]TcMAA to act as a predictive pre-treatment surrogate for dose distribution, which should be administered during work-up angiography using an appropriate catheter position and delivery technique to improve reproducibility [13–15, 21, 22]. Subsequently, [^99m^Tc]TcMAA activity distribution may serve as an input for dosimetry analysis. Some studies defined tumors on anatomic imaging and calculated prescribed absorbed doses after co-registration with [^99m^Tc]TcMAA SPECT, while other studies defined tumors directly on [^99m^Tc]TcMAA SPECT by using an activity-isocontour threshold [1, 2]. On one hand, the latter method has the advantage of omitting the need to co-register anatomic images with [^99m^Tc]TcMAA, which is prone to error. On the other hand, [^99m^Tc]TcMAA threshold segmentation may incorrectly define VOI boundaries based on operator-dependent thresholding. Because different studies use different dosimetry methods, comparison of results between studies and dose guidance may be hampered. Currently, it is not well understood how these segmentation methods compare on a patient-level and what implications this may have for clinical outcomes. Here, we present results from the TARGET study with a focus on real-world data to evaluate the clinical utility and reproducibility of two segmentation approaches. The two segmentation methods are based on anatomy using diagnostic CT/MRI imaging or [^99m^Tc]TcMAA accumulation using [^99m^Tc]TcMAA SPECT imaging for segmentation of tumor and normal tissue volumes in patients with HCC undergoing glass ^90^Y-microspheres radioembolization.

## Materials and methods

### Study design

The TheraSphere^™^ Advanced Dosimetry Retrospective Global Study Evaluation in Hepatocellular Carcinoma Treatment (TARGET) study was an international, multi-center, retrospective, single-arm study of patients from 13 centers in eight countries treated using glass ^90^Y-microspheres for HCC [1].

Sites received approval of the protocol from their Institutional Review Boards (IRBs) and/or Independent Ethics Committees (IECs). Patients who met study eligibility criteria were enrolled at each site in consecutive reverse chronological order. Selection of all consecutive eligible patients minimizes selection bias versus patients selected in other ways. All patients were treated between January 1st, 2010 and December 31st, 2017 according to standard practice outlined in the *T*heraSphere^™^ instruction for use [23] and local procedures.

The primary aim of this study was to establish dose-efficacy relationships by associating TAD to clinical outcomes (i.e., objective response rates (ORR) and overall survival (OS)) for two distinct methods: (1) anatomic tumor segmentation on MRI/CT, (2) [^99m^Tc]TcMAA threshold-based tumor segmentation on SPECT. A secondary aim was to compare the agreement between pre-treatment [^99m^Tc]TcMAA SPECT and post-treatment ^90^Y PET/CT absorbed dose distribution.

### Inclusion and exclusion criteria

The key patient inclusion criteria in this retrospective analysis were: adults (≥ 18 years of age) with liver-dominant disease; at least one tumor ≥ 3 cm, with or without portal vein tumor thrombosis; Child-Pugh stage A or B7; BCLC stage A, B, or C. Additionally, patients were required to have had diagnostic imaging consisting of multi-phase contrast enhanced CT or contrast enhanced MRI within three months prior to [^99m^Tc]TcMAA SPECT or SPECT/CT imaging; received an infusion of [^99m^Tc]TcMAA and glass ^90^Y-microspheres from a single location sufficient to cover the tumor(s) based on angiography. Patients were ineligible if they had prior external beam radiation treatment to the liver; prior loco-regional liver-directed therapy (e.g., trans-arterial chemoembolization) and/or radioembolization; prior liver resection or transplantation; anti-cancer therapy between first radioembolization treatment and three-month imaging; hepatic vein invasion; and administration to ≤ 2 segments (i.e., radiation segmentectomy).

### Data collection

Patient data were collected through a retrospective review of medical records. Imaging data were collected at baseline (+/− 30 days), 42 days (+/− 17 days), 90 days (+/− 30 days), 180 days (+/− 30 days) after treatment; any additional long-term imaging data available between 180 and 400 days was also collected. Treatment-specific variables included details of the preparation and imaging of [^99m^Tc]TcMAA, administration of [^99m^Tc]TcMAA and glass ^90^Y-microspheres, and patient treatment efficacy and safety outcomes (i.e., tumor response performed by a board-certified radiologist not involved in dosimetric analysis according to mRECIST and RECIST 1.1, overall survival in months and toxicity grades according to CTCAE version 4.02). ORR was reported as best response over all scans during follow-up.

### Imaging and dosimetry

Imaging for [^99m^Tc]TcMAA SPECT or SPECT/CT imaging or post ^90^Y PET was based on standard institutional practice. Enrolled subjects required angiography documenting the catheter position for [^99m^Tc]TcMAA and glass ^90^Y-microspheres administration, diagnostic contrast enhanced imaging (CT or MRI) and [^99m^Tc]TcMAA SPECT or SPECT/CT imaging by two-headed SPECT camera system. The catheter position was documented per protocol, either selective or lobar, with most patients undergoing lobar infusion. Dosimetry analysis consisted of image co-registration (i.e., CT/MRI and [^99m^Tc]TcMAA SPECT/CT), segmentation of volumes, and absorbed dose calculation based on multicompartment dosimetry. The co-registration was adjusted as needed using software tools with the clinician as the arbiter of co-registration quality before dosimetry calculations were performed. Pre-treatment volumes and absorbed doses were calculated using Simplicit^90^Y dosimetry software version 1.1 (Mirada Medical Ltd., Oxford, United Kingdom).

Segmentation involved whole liver, whole liver normal tissue, perfused liver, perfused normal liver (i.e., perfused liver minus sum of all tumors ≥ 2 cm), and total perfused tumors (i.e., sum of all tumors ≥ 3 cm). An independent board-certified radiologist at each site, who was not involved in assessment of tumor response, inspected all segmented volumes to confirm that the volume corresponded to the correct description and evaluated the quality of the co-registration.

Image segmentation was performed according to two distinct methods. For the anatomic method, segmentation of whole liver volume, perfused volume(s), and tumor volume(s) was based on anatomic boundaries as visualized on the diagnostic CT or MR. For the [^99m^Tc]TcMAA threshold method, whole liver volume was segmented using anatomic imaging, while the perfused volume and tumor volume segmentation was based on an activity-isocontour threshold that was deemed to best delineate the volumes of interest per the clinician. The [^99m^Tc]TcMAA threshold method was facilitated with the ‘% threshold’ tool within Simplicit^90^Y for segmentation and was applied within a user-defined box around the desired volume as visualized on [^99m^Tc]TcMAA SPECT/CT. Manual adjustments to the isocontour based VOI to maintain volumes within liver or perfused volume were allowed. The anatomic method relied solely on anatomic segmentation on MRI or CT of the individual volumes of interest.

All [^99m^Tc]TcMAA counts within the perfused volume were assigned to the patient specified activity. Dose calculation was performed using the VOI and the counts from the co-registered [^99m^Tc]TcMAA SPECT/CT based on the MIRD schema using a patient relative calibration factor and the local deposition method.

In a subset of patients, for whom both pre-treatment [^99m^Tc]TcMAA SPECT and post-treatment ^90^Y PET/CT data were available, dosimetry was also performed on post-treatment ^90^Y PET/CT. The anatomic method was used for this purpose, co-registering baseline CT/MRI (including all segmentations used for pre-treatment analysis) to the ^90^Y PET/CT.

### Statistics

Demographic data, race and ethnicity, and other baseline characteristics were summarized. Continuous data were summarized with means, medians, standard deviations, minima, and maxima. Categorical data were summarized with observed counts and percentages for each category. Normal Tissue Complication Probability (NTCP) and Tumor Control Probability (TCP) regression curves were fitted using logistic regression. NTCP curves were fitted to assess the relationship between the probability of grade 3 or higher hyperbilirubinemia and the perfused liver NTAD. In addition, NTCP curves were fitted to assess the relationship between the probability of dose related adverse events (AEs) of interest (i.e., hyperbilirubinemia, ascites, pain, fatigue, nausea, and post-embolization syndrome) and the perfused liver NTAD. The TCP curve showed the relationship between the probability of achieving an ORR (according to mRECIST and separately according to RECIST 1.1) and the total perfused TAD. Kaplan-Meier methodology was used to analyze OS. Cox regression was used to evaluate the relationship between TAD and OS. A multivariable logistic regression was also used to assess the relationship between the occurrence of grade 3 or higher hyperbilirubinemia and perfused volume NTAD after taking account pre-defined covariates of interest, according to the following steps: (1) Each covariate was included, one at a time, together with NTAD, in a series of univariable models. If the covariate had 2-sided *p*-value < 0.1, it was included in the model selection procedure described in Step 2; (2) A backward elimination procedure was used to determine the final multivariable model, starting with all the significant covariates identified in Step 1. A 2-sided significance level of 10% was used for a covariate to remain in the model. The process was repeated until all the remaining covariates had 2-sided *p*-values < 0.1. Bland-Altman analysis, including calculation of the 95% limits of agreement, was performed by plotting the difference in absorbed dose (pre-treatment imaging minus post-treatment imaging) against the post-treatment imaging absorbed dose. Pearson’s correlation coefficient was calculated, and scatter plots were provided for pre- and post-treatment imaging absorbed doses to normal tissue and tumor respectively.

## Results

A total of 209 patients met the inclusion criteria and were included in the study. Baseline patient characteristics are included in Table 1. The majority of treated HCC patients had unilobar disease (70.8%), a solitary lesion (69.4%) of at least 5 cm (80.4%), and Child-Pugh A (89.5%). The median total perfused liver absorbed dose was 117.3 Gy (range: 27.1, 286.1) by anatomic segmentation.

**Table 1:** Baseline characteristics

Patient characteristics	Treated population (*N* = 209) *N* (%)
Median age (range), years	66 (27–87)
Gender, male	166 (79.4%)
Etiology*	
Hepatitis B	31 (14.8%)
Hepatitis C	69 (33.0%)
Alcohol	48 (23.0%)
Non-alcoholic steatohepatitis	20 (9.6%)
Liver disease of unknown etiology	32 (15.3%)
Other	28 (13.4%)
Cirrhosis	185 (88.5%)
ECOG status	
0	135 (64.6%)
1	67 (32.1%)
≥2	7 (3.3%)
BCLC status	
A	27 (12.9%)
B	68 (32.5%)
C	114 (54.5%)
Child-Pugh status	
A(5–6)	187 (89.5%)
B7	22 (10.5%)
Unilobar or bilobar disease	
Unilobar	148 (70.8%)
Bilobar	61 (29.2%)
With PVT	69 (33.0%)
Largest lesion diameter (RECIST 1.1)	
≥3 to < 5 cm	41 (19.6%)
≥5 to < 8 cm	72 (34.4%)
≥8 cm	96 (45.9%)
Total number of lesions	
1	145 (69.4%)
2	45 (21.5%)
3	14 (6.7%)
4–10	5 (2.4%)

*Patients could have multiple etiologies

Volumes and absorbed doses by anatomic or [^99m^Tc]TcMAA threshold methods are included in Table 2. Total perfused tumor and perfused normal tissue volumes were larger by the anatomic compared to the [^99m^Tc]TcMAA threshold method, resulting in differences in absorbed dose [24]. 

**Table 2:** Volumes and absorbed doses

Method	[^99m^Tc]TcMAA SPECT	Anatomic
**Total perfused tumor volume (cm**^**3**^), ***n***	209	209
Mean (standard deviation)	235.7 (224.1)	307.1 (322.7)
Median (Min, Max)	181.4 (7.5, 1366.7)	204.8 (7.9, 1688.5)
**Total perfused tumor absorbed dose (Gy)**, ***n***	209	209
Mean (standard deviation)	373.9 (283.4)	254.5 (166.4)
Median (Min, Max)	302.0 (32.0, 2468.7)	216.0 (14.0, 1130.4)
**Total perfused normal tissue volume (cm**^**3**^), ***n***	207	208
Mean (standard deviation)	732.0 (475.6)	873.6 (429.5)
Median (Min, Max)	627.9 (5.0, 2477.3)	838.0 (109.0, 2495.7)
**Total perfused normal tissue absorbed dose (Gy)**, ***n***	207	208
Mean (standard deviation)	123.4 (84.9)	92.45 (42.9)
Median (Min, Max)	103.4 (14.8, 533.9)	87.3 (16.8, 270.1)
**Whole liver normal tissue volume (cm**^**3**^), ***n***	207	209
Mean (standard deviation)	1866.2 (618.8)	1626.5 (554.6)
Median (Min, Max)	1731.1 (799.2, 3856.7)	1514.2 (627.9, 3600.0)
**Whole liver normal tissue absorbed dose (Gy)**, ***n***	206	208
Mean (standard deviation)	39.5 (20.6)	48.9 (24.5)
Median (Min, Max)	37.6 (0.3, 148.5)	48.1 (5.4, 166.0)

CT = computerized tomography; [^99m^Tc]TcMAA = Technetium-99m macroaggregated albumin; MRI = magnetic resonance imaging; n = number of patients with data; SPECT = single-photon emission computed tomography. Some of the data was missing in three patients

Per the above, median total perfused TAD was higher for the [^99m^Tc]TcMAA threshold method, 302.0 Gy, versus the anatomic method, 216.0 Gy. Median NTAD was also higher for the [^99m^Tc]TcMAA threshold method, 103.4 Gy, versus the anatomic method, 87.3 Gy. Whole liver NTAD (i.e., including perfused and non-perfused normal tissue) was lower for the [^99m^Tc]TcMAA threshold method, 37.6 Gy, versus the anatomic method, 48.1 Gy.

ORR was 61.7% (129/209; 95% CI: 55.0%, 68.0%) by mRECIST and 34.4% (72/209; 95% CI: 28.3%, 41.1%) by RECIST 1.1. Median OS was 20.3 months (95% CI: 16.7, 26.4). A dose-effect relationship was found regardless of the segmentation method used to calculate TAD. For mRECIST ORR, odds ratio per 100 Gy TAD increase was 1.22 (95% CI: 1.01–1.49; *p* = 0.044) for the anatomic method and 1.19 (95% CI: 1.04–1.37; *p* = 0.012) for the [^99m^Tc]TcMAA threshold method. For RECIST 1.1 ORR, odds ratio per 100 Gy TAD increase was 1.21 (95% CI: 1.02–1.44; *p* = 0.030) for the anatomic method and 1.13 (95% CI: 1.01–1.26; *p* = 0.028) for the [^99m^Tc]TcMAA threshold method. For OS, the hazard ratio per 100 Gy TAD increase was 0.826 (95% CI: 0.714–0.954; *p* = 0.009) and 0.847 (95% CI: 0.765–0.936; *p* = 0.001) by anatomic and [^99m^Tc]TcMAA threshold methods, respectively. NTCP curve fitting, and multivariate modeling did not find a relationship between perfused volume NTAD and grade 3 or higher hyperbilirubinemia (Supplementary Tables 1 and 2).

**Fig. 1:**
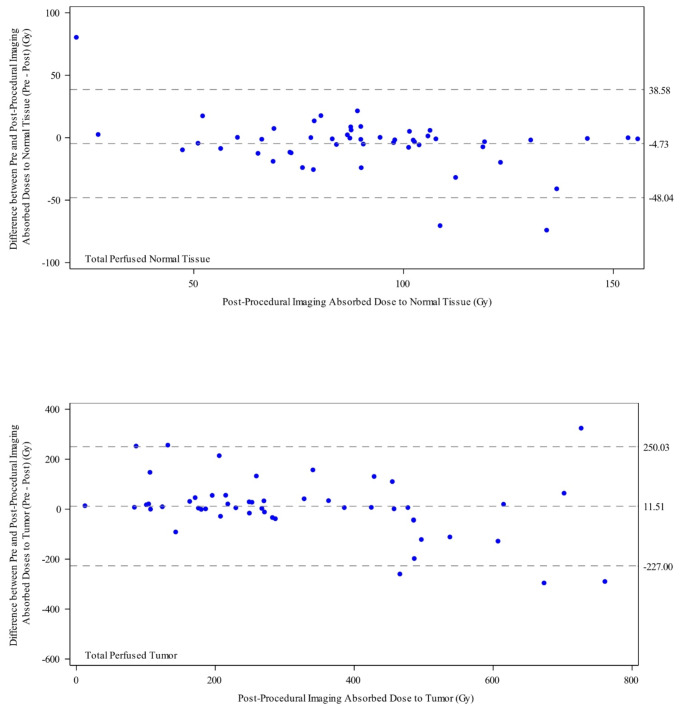
Bland-Altman plot of pre- and post-treatment total perfused normal tissue (bias − 4.7 Gy with 95% limits of agreement − 48.0 to 38.6 Gy) (**A**) and total perfused tumor (bias 11.5 Gy with 95% limits of agreement − 227.0 to 250.0 Gy) (**B**). The center horizontal line shows the bias, and the horizontal lines above and below the center line shows the 95% limits of agreement

The relationship between pre-treatment and post-treatment TAD and NTAD was assessed in the subgroup of patients that had both pre- and post-treatment imaging performed (Table 3). According to Bland-Altman analysis comparing pre-treatment [^99m^Tc]TcMAA SPECT (anatomic segmentation) and post-treatment ^90^Y PET/CT, the bias for TAD was + 11.5 Gy (i.e., on average pre-treatment TAD was 11.5 Gy greater than the post-treatment value), with 95% limits of agreement of -227.0 to 250.0 (Fig. 1). There was a strong positive relationships between pre- and post-treatment TAD (Pearson’s correlation coefficient = 0.80) (Supplementary Table 3, Supplementary Figs. 1 and 2). The bias for total perfused NTAD was − 4.7 Gy with 95% limits of agreement of -48.0 to 38.6 (Fig. 1) and for whole liver NTAD the bias was − 1.9 Gy with 95% limits of agreement of -29.9 to 26.2. There was a strong positive relationship between pre- and post-treatment whole liver NTAD (Pearson’s correlation coefficient = 0.72), and for total perfused NTAD (Pearson’s correlation coefficient = 0.71).

**Table 3:** Subset of 52/209 patients for whom post-treatment PET/CT was available

Method	[^99m^Tc]TcMAA SPECT/CT	^90^Y PET/CT
**Total perfused tumor absorbed dose (Gy)**, ***n***	52	52
Mean (standard deviation)	325.65 (185.1)	314.1 (188.9)
Median (Min, Max)	279.3 (26.1, 1050.9)	263.1 (12.4, 760.8)
**Total perfused normal tissue absorbed dose (Gy)**, ***n***	51	51
Mean (standard deviation)	86.35 (28.2)	91.1 (28.7)
Median (Min, Max)	92.2 (29.8, 154.9)	89.8 (22.0, 155.8)
**Whole liver normal tissue absorbed Dose (Gy)**, ***n***	51	51
Mean (standard deviation)	52.4 (19.3)	54.3 (18.5)
Median (Min, Max)	54.1 (9.7, 83.8)	56.8 (11.1, 86.8)

[^99m^Tc] = technetium-99m; CT = computerized tomography; MAA = macroaggregated albumin; SPECT = single-photon emission computed tomography; PET = positron emission tomography. Data on whole liver and perfused volume NTAD was missing for one patient. [^99m^Tc]TcMAA SPECT and ^90^Y PET/CT data were based on anatomic segmentation

**Fig. 2:**
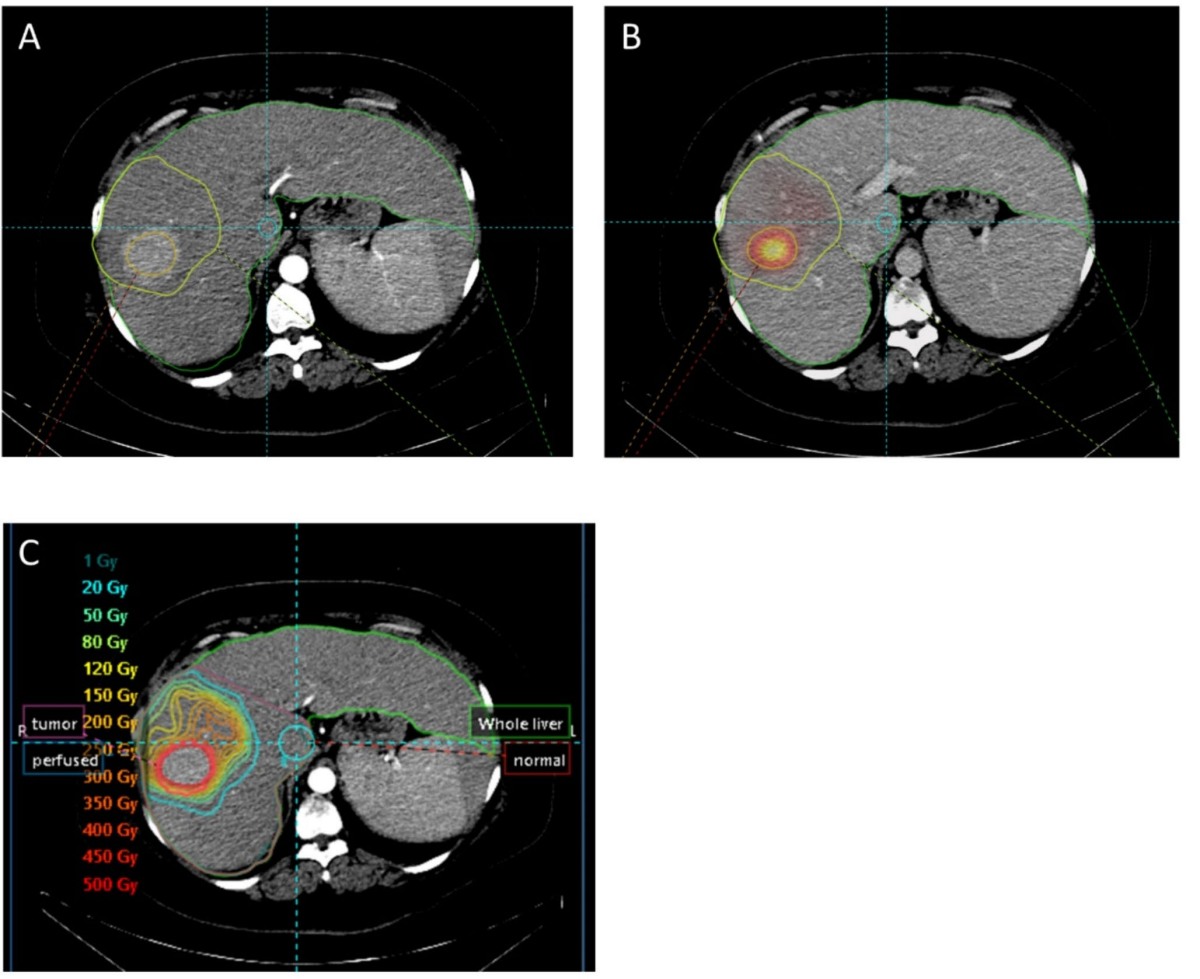
In most cases, after proper co-registration, the hypervascular tumor segmented on CT/MRI (**A**) closely matches the high focal accumulation of [^99m^Tc]TcMAA on SPECT/CT (**B**), resulting in high tumor-absorbed doses and sharp dose drops in surrounding liver tissue (**C**). Both segmentation methods will be accurate

**Fig. 3:**
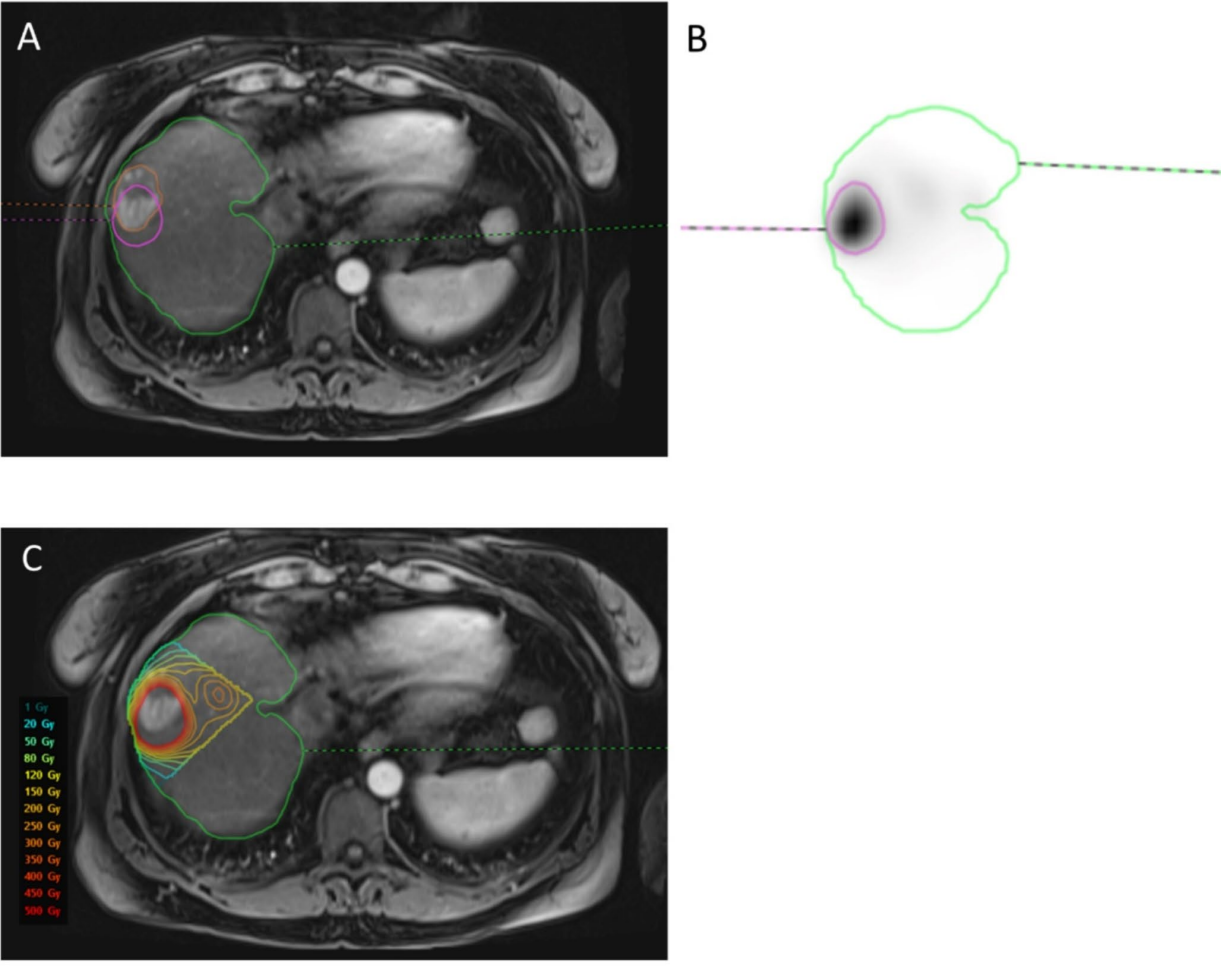
The hypervascular lesion in segment 8 (**A**) does not align with the high focal [^99m^Tc]TcMAA accumulation on SPECT (**B**; purple color), favoring the [^99m^Tc]TcMAA threshold segmentation method to capture the most accurate tumor absorbed dose (**C**)

**Fig. 4:**
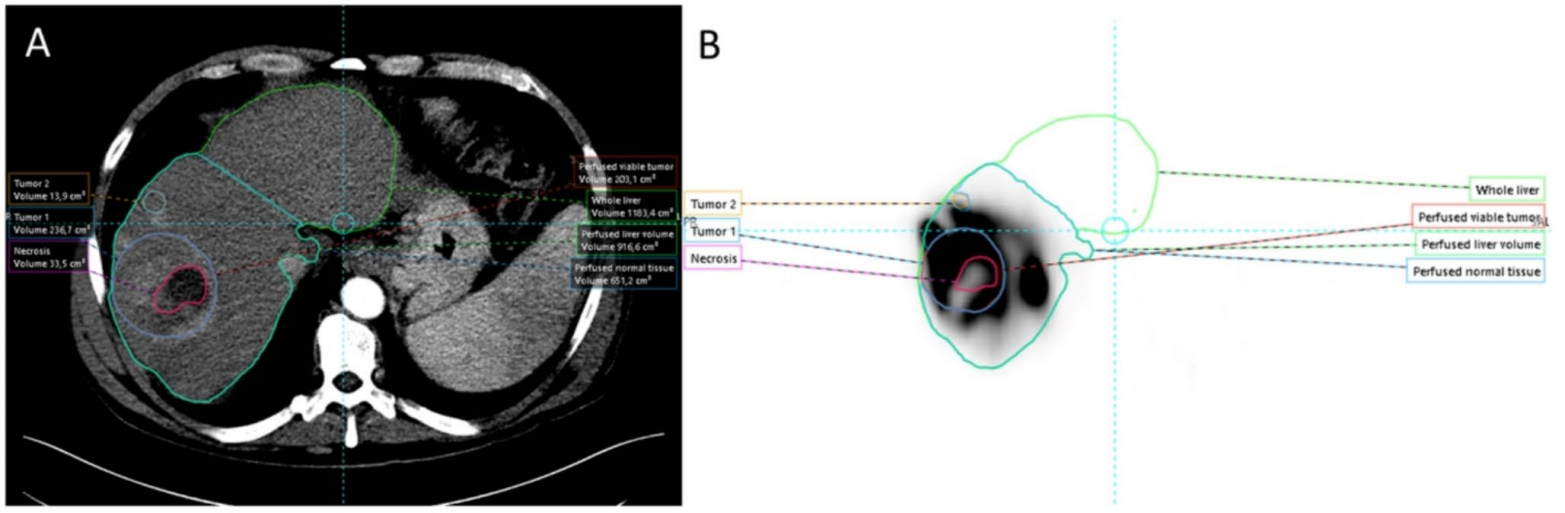
The tumor segmented on CT (**A**; blue color, central necrosis in red color) does not align with the more heterogeneous [^99m^Tc]TcMAA accumulation in and around the tumor (**B**). This limits the [^99m^Tc]TcMAA threshold segmentation method in this case, favoring the anatomic segmentation method

Both anatomic and [^99m^Tc]TcMAA threshold segmentation methods allowed dosimetric analysis. Figure 2 demonstrates a case where both segmentation methods were substantially equivalent. Tumor volume closely matched for the anatomic and [^99m^Tc]TcMAA threshold method with high TAD and sharp drops in counts in the surrounding liver tissue. In Fig. 3 the hypervascular tumor on MRI is misaligned with [^99m^Tc]TcMAA SPECT. Misregistration between the [^99m^Tc]TcMAA threshold and anatomic segmentation of the tumor on CT or MRI will result in a less accurate TAD and may affect NTAD values. Because in this case the tumor can be clearly defined on [^99m^Tc]TcMAA SPECT, using the [^99m^Tc]TcMAA threshold segmentation method obviates the need for (erroneous) registration with MRI. In the final example, anatomic tumor segmentation of a well-defined tumor on CT does not match the activity distribution on [^99m^Tc]TcMAA SPECT (Fig. 4). Because in this case the tumor cannot be clearly defined on [^99m^Tc]TcMAA SPECT it seems best to stick with the anatomic segmentation method and accept potential errors in registration between CT and [^99m^Tc]TcMAA SPECT. These examples illustrate the need for an individual approach and the importance of selecting the segmentation method that best fits with available imaging.

## Discussion

The TARGET study provides real-world data confirming a significant dose-effect relationship between TAD and ORR and between TAD and OS in HCC patients treated with glass ^90^Y-microspheres [1]. Increasing TAD resulted in increased probability of tumor response and longer OS with an acceptable toxicity profile. The comparable performance of two distinct segmentation methods that were previously reported in multiple publications (i.e., anatomic and [^99m^Tc]TcMAA threshold segmentation methods) confirmed the validity of using either method [2, 13–15]. Although these methods lead to individual differences in terms of volumes and resulting absorbed doses, overall, the association with clinical outcomes was preserved regardless of method used. In the TARGET patients (*n* = 209), the two segmentation methods were compared and [^99m^Tc]TcMAA SPECT segmentation gave, on average, TAD 43.9% higher/NTAD 21.3% higher than anatomic segmentation by Bland-Altman analysis [24].

In clinical practice, most HCC patients can be evaluated with the anatomic method, because most HCC lesions are relatively hypervascular and can be easily delineated on contract enhanced diagnostic CT or MR. After co-registration with SPECT/CT, the counts from SPECT in the tumor volume can be translated in a prescribed TAD. However, select patients may be better evaluated with the [^99m^Tc]TcMAA threshold method. The lower dose threshold is adjusted to expand/reduce the VOI boundaries to appropriately incorporate the total perfused tumor as noted in the following examples: (1) in case of misalignment of the anatomic tumor volume and focal tumor activity on SPECT, (2) in case of an ill-defined tumor volume on anatomic images, but demarcated tumor activity on SPECT. On the other hand, in case of high and focal [^99m^Tc]TcMAA activity in the normal liver tissue, [^99m^Tc]TcMAA thresholding is not feasible, and the anatomic method is clearly preferred. A hybrid approach where the anatomic method is the primary source of information with consultation of [^99m^Tc]TcMAA threshold method estimated distribution may ultimately improve pre-treatment dosimetry estimation. In case both methods fail (e.g., diffuse infiltrative disease), multicompartment dosimetry is not possible and single compartment modeling should be used.

Published clinical studies demonstrate that TAD can predict tumor response and NTAD can predict safety/tolerability [2, 12–15, 22, 25]. The DOSISPHERE-01 study, a prospective, randomized, controlled study using multicompartment dosimetry based on [^99m^Tc]TcMAA SPECT/CT imaging with specific TAD and NTAD targets, demonstrated that this treatment approach was superior to single compartment dosimetry (i.e., 120 ± 20 Gy average absorbed dose to the perfused volume) for tumor response rate three months after treatment, 71.4% versus 35.7% respectively (according to European Association for the Study of the Liver (EASL) criteria), with a doubling in median OS from 10.7 months (95% CI: 6.0, 14.8) to 26.6 months (95% CI: 11.7, not reached), hazard ratio 0.38 [95% CI: 0.19, 0.82] and a preserved safety profile [11]. In contrast with TARGET, the [^99m^Tc]TcMAA threshold method was used for segmentation and dosimetry. Reported absorbed doses in DOSISPHERE ([^99m^Tc]TcMAA threshold method) can therefore not directly be compared with those reported in TARGET [1] (anatomic method) but can be compared with TARGET when using the [^99m^Tc]TcMAA threshold method.

Select studies evaluated the agreement between [^99m^Tc]TcMAA imaging and post-treatment ^90^Y PET/CT imaging [13–15, 22, 25]. In a comparable cohort of 23 HCC patients (unilobar 74%, Child-Pugh A 91%, median tumor volume 353 cm^3^), pre-treatment [^99m^Tc]TcMAA SPECT dosimetry was highly correlated and concordant with ^90^Y microspheres PET dosimetry for TAD (Pearson’s correlation coefficient = 0.87) and for NTAD (Pearson’s correlation coefficient = 0.91) [15]. Bland-Altman analysis showed acceptable bias (close to zero) and 95% limits of agreement for both TAD (ca. − 50 to − 40 Gy) and NTAD (ca. − 13 to 12 Gy). Interestingly, catheter positioning proved to have a significant influence on the found agreement, both the catheter position between [^99m^Tc]TcMAA procedure and ^90^Y, and the catheter position in relation to major bifurcations [15, 26]. The TAD difference between [^99m^Tc]TcMAA and ^90^Y was lower when the catheter position was identical than when it differed (16 Gy vs. 37 Gy, *p* = 0.007). Besides, dose differences were negatively correlated with the catheter tip distance from major artery bifurcation [15]. It is likely that the hypervascular nature of HCC and the large size of the tumors contributed to found agreement, which confirms the use of [^99m^Tc]TcMAA in this particular setting.

Limitations of this study include the retrospective collection of the data, although the robust sample size and global site locations is important for generalizability and to further optimize the use of glass ^90^Y-microspheres in the real-world setting. BCLC stage was site reported, and for patients with an ECOG score ≥ 1, it is challenging in a retrospective study to define if ECOG status is disease related or not. There are limitations with the Bland-Altman analysis conducted in this study. First, the patients included in this analysis only represent 25% of the total population. Second, the parameters and techniques necessary for a reliable assessment of pre-treatment versus post-treatment agreement, to more accurately predict TAD and NTAD, were not uniformly incorporated in treatment planning. These parameters include identical type and location of catheter for [^99m^Tc]TcMAA and glass ^90^Y-microspheres infusion and similar infusion pressure for [^99m^Tc]TcMAA and glass ^90^Y-microspheres. As a result, the 95% limits of agreement for TAD and NTAD within the TARGET study were wider than for other studies recently published [14, 15]. Nevertheless, a statistically significant association between TAD and efficacy parameters was observed.

Although the anatomic method for segmentation seems more intuitive, in individual cases, the [^99m^Tc]TcMAA threshold method or a combination of the two methods may be more practical. However, doses will be different between methods. This is important to consider when analyzing study results and when implementing specific dose thresholds in clinical practice. Future studies should explicitly report on used methods to further optimize individualized treatment planning based on dosimetry.

In conclusion, real-world data support both [^99m^Tc]TcMAA imaging threshold-based segmentation and anatomical segmentation to estimate NTAD and TAD prior to treatment with glass ^90^Y-microspheres. Both anatomic and [^99m^Tc]TcMAA threshold methods were suitable for accurate treatment planning. However, significant differences between both methods exist that should be accounted for when comparing study results.

## Electronic supplementary material

Below is the link to the electronic supplementary material.

Supplementary Material 1

## Abbreviations

HCC: Hepatocellular carcinoma
^90^Y: [90Y]yttrium
TAD: Tumor absorbed dose
NTAD: Normal tissue absorbed dose
[^99m^Tc]TcMAA: Technetium-99 macroaggregated albumin
SPECT/CT: Single photon emission computed tomography/computed tomography
PET/CT: Positron emission tomography/computed tomography
MIRD: Medical internal radiation dose
PVT: Portal vein thrombosis
TARGET: The TheraSphere™ Advanced Dosimetry Retrospective Global Study Evaluation in Hepatocellular Carcinoma Treatment
AE: Adverse event
SAE: Serious adverse event
OS: Overall survival
RECIST: Response Evaluation Criteria in Solid Tumors
BCLC: Barcelona Clinic Liver Cancer
ECOG: Eastern Cooperative Oncology Group
CR: Complete response
PR: Partial response
AFP: Alpha fetoprotein
CTCAE: Common Terminology Criteria for Adverse Events
MRI: Magnetic resonance imaging
VOI: Volume of interest
ORR: Objective response rate
mRECIST: Modified Response Evaluation Criteria in Solid Tumors
EASL: European Association for the Study of the Liver
NTCP: Normal Tissue Complication Probability
TCP: Tumor Control Probability
