# Supplementary material for: Utility of pre-procedural [99mTc]TcMAA SPECT/CT Multicompartment Dosimetry for Treatment Planning of 90Y Glass microspheres in patients with Hepatocellular Carcinoma: comparison of anatomic versus [99mTc]TcMAA-based Segmentation
Source: Eur J Nucl Med Mol Imaging. 2024 Sep 27;52(2):744–55. doi: 10.1007/s00259-024-06920-6 (PMC11732885; doi:10.1007/s00259-024-06920-6)
Supplement: Supplementary file 1 — Supplementary Material 1 [file 259_2024_6920_MOESM1_ESM.docx]

**Supplementary Table 1.** Normal Tissue Complication Probability (NTCP) analysis (n=209).

| **Dosimetry method** | **Odds Ratio** | **95% CI** | **p-value** |
| --- | --- | --- | --- |
| MRI or CT  (Anatomic method) | 1.03 | 0.90, 1.18 | 0.634 |
| [^99m^Tc]TcMAA SPECT | 0.93 | 0.83, 1.05 | 0.239 |

[^99m^Tc] = technetium-99m; CI = confidence interval; CT = computerized tomography; MAA = macroaggregated albumin; MRI = magnetic resonance imaging; SPECT = single-photon emission computed tomography; Note: Logistic regression of bilirubin >3 times upper limit of normal in absence of disease progression versus absorbed dose to perfused volume normal tissue. Odds ratio for absorbed dose corresponds to a 10 Gy increase in absorbed dose.

**Supplementary Table 2.** Multivariate modelling of Normal Tissue Complication Probability (NTCP) and ≥grade 3 hyperbilirubinemia.

| **Dosimetry method** | **Parameter** | **Odds Ratio** | **95% CI** | **p-value** |  |
| --- | --- | --- | --- | --- | --- |
| **Total perfused normal tissue** | | | | | |
| Anatomic | Absorbed dose (Gy) | 1.11 | 0.94, 1.29 | 0.214 |  |
|  | Baseline bilirubin  <1.0 mg/dL vs. ≥1.0 mg/dL | 0.23 | 0.06, 0.94 | 0.041 |  |
|  | PVT [^99m^Tc]TcMAA targeting  No PVT targeting vs. PVT targeting  No PVT vs. PVT targeting | 8.85  1.64 | 0.86, 91.60  0.18, 14.80 | 0.048 |  |
| **Total perfused normal tissue** | | | | | |
| [^99m^Tc]TcMAA SPECT | Absorbed dose (Gy) | 0.94 | 0.83, 1.06 | 0.312 |  |
|  | Baseline bilirubin  <1.0 mg/dL vs. ≥1.0 mg/dL | 0.31 | 0.08, 1.16 | 0.083 |  |

[^99m^Tc] = technetium-99m; CI = confidence interval; CT = computerized tomography; MAA = macroaggregated albumin; MRI = magnetic resonance imaging; SPECT = single-photon emission computed tomography; Note: Logistic regression of bilirubin >3 times upper limit of normal in absence of disease progression versus absorbed dose to normal tissue. Odds ratio for absorbed dose corresponds to a 10 Gy increase in absorbed dose.

**Supplementary Table 3.** Bland-Altman analysis of agreement and Pearson’s correlation coefficient of pre-treatment [^99m^Tc]TcMAA SPECT (anatomic method) and post-treatment ^90^Y PET/CT.

| **Volume of interest** | **Bias (95% limits of agreement), Gy** | **Pearson’s Correlation Coefficient** |
| --- | --- | --- |
| Total perfused normal tissue | -4.7 (-48.0, 38.6) | 0.71 |
| Whole liver normal tissue | -1.9 (-29.9, 26.2) | 0.72 |
| Total perfused tumor | 11.5 (-227.0, 250.0) | 0.80 |

**Supplementary Figure 1.** Scatter plot of pre- and post-treatment absorbed doses to total perfused normal tissue. Pearson’s correlation coefficient was 0.71.


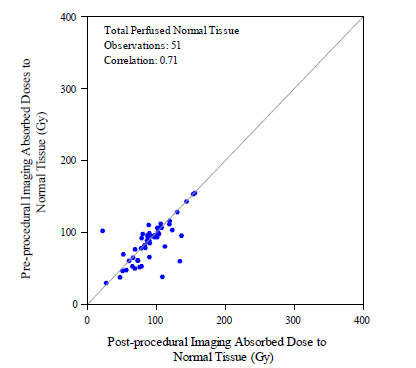


Figure 4.

**Supplementary Figure 2.** Scatter plot of pre- and post-treatment absorbed doses to total perfused tumor. Pearson’s correlation coefficient was 0.80.

**
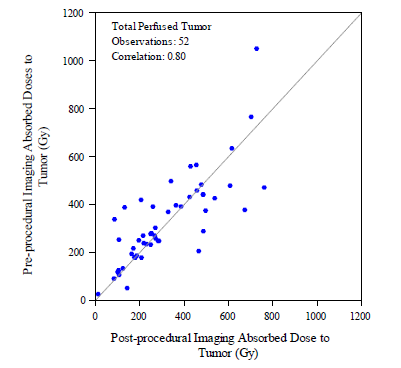
**
